# The Serum Cytokines' Network and Th1/Th2 Profile Balance in patients with Chronic Urticaria

**DOI:** 10.1186/2045-7022-5-S1-O7

**Published:** 2015-03-11

**Authors:** Muataz Ali Hamad, Nunu Mitskevich, Ketevan Machavariani

**Affiliations:** 1 Petre Shotadze Tbilisi Medical Academy, Immunology Department, Tbilisi; Georgia; 2 Iv. Javakhishvili Tbilisi State University, Immunology and Microbiology Department, Tbilisi; Georgia; 3 Tbilisi State Medical University, Allergy and Clinical Immunology Department, Tbilisi, Georgia

## Background

Chronic urticaria is a skin disease with an important immunologic profile. There is a great interest not only due to its special clinical manifestation, but also due to its pathogenic mechanism. New data support the participation of ILs (interleukins) in the pathophysiology of CU (chronic urticaria). The aim of this study is to explore the change and its significance of cytokines, comparative characteristics of immune response - Th1/Th2 cytokines profiles balance in patients with CU in relation to atopy.

## Method

This prospective study included 46 patients with CU and 12 patients without urticaria or angioedema as a control group. Patients diagnosed with CU were separated to two subgroups with and without atopy (14/32). These patients were randomly selected at the Center of Asthma, Allergy and Clinical Immunology at TSMU. In peripheral blood was detected T CD4+, CD8+lymphocytes by flow cytometry using Becton Dikinson's monoclonal antibodies. The serum levels of IgE (total), sIL-2R, IL-6, IFN-γ, TNF-α, IL-4, IL-5, IL10, IL-13 were measured using ELISA, R&D System kits, Germany, at the lab of Immunology and Microbiology at TSU.

## Results

The concentrations of sIL-2R, IL-6, TNF-α, IFN-γ in CU patients were significantly higher than in control group (p=0.01). sIL-2R, IL-6, TNF-α concentrations in the atopic patients were higher than those in the CU patients without atopy (p<0.05). IFN-γ levels did not differ depending on the presence of atopy (p=0.9). IL-4, IL-5, IL10, IL-13 levels were higher in subjects with a history of angioedema (18.6pg/ml(1;31.7) compared with those without this condition (0pg/ml(0;22.6). This difference was statistically significant (Mann-Whitney test, p=0.01).Cytokine levels that have been studied correlated with each other as it follows – IFN-γ levels correlated with IL-10 and IL-13 levels (p=0.05), IL5-levels correlated with IL-6 levels (p<0.001), and other statistically significant correlations between the eight cytokines have not been observed in patients with CU.

## Conclusion

According our study was shown that chronic urticaria is not specifically characterized only by increasing level of Th1 or Th2 profile cytokines. There is a combination of mixed Th1/Th2. Not all patients with CU have the same profile of ILs. There was a dominant Th1 response in CU patients without atopy. Total Ig E level and certain etiological factors relatively specifically modulate the immune response of CU.

